# Fecal and urinary incontinence in older men with hip fracture: a geriatric syndrome with prognostic significance

**DOI:** 10.1007/s41999-026-01624-6

**Published:** 2026-10-07

**Authors:** Aino T. Hellman-Bronstein, Tiina H. Luukkaala, Seija S. Ala-Nissilä, Maria S. Nuotio

**Affiliations:** 1 Department of Geriatric Medicine, Turku University Hospital and University of Turku, Turku, Finland; 2 Research, Development and Innovation Center, Tampere University Hospital, Tampere, Finland; 3 Health Sciences, Faculty of Social Sciences, Tampere University, Tampere, Finland; 4 Department of Obstetrics and Gynecology, Turku University Hospital and University of Turku, Turku, Finland; 5 Department of Geriatric Medicine, Seinäjoki Central Hospital, Seinäjoki, Finland

**Keywords:** Urinary incontinence, Fecal incontinence, Hip fracture, Men, Older adults

## Abstract

**Purpose:**

Hip fracture in older men is associated with disproportionately poor outcomes compared to women, yet the prevalence and prognostic significance of incontinence in this population remain understudied. This study aimed to determine the prevalence of urinary (UI) and fecal incontinence (FI) in older men with hip fracture, evaluate their association with 1-year mortality, and identify factors linked to UI at post-fracture out-patient comprehensive geriatric assessment (CGA).

**Methods:**

This retrospective study analyzed data from 644 men aged ≥65 with first hip fracture (2007–2019) at Seinäjoki Central Hospital, Finland. Cox proportional hazards regression assessed associations of baseline UI (yes/no) and FI (yes/no) with 1-year mortality (*n*=549). Binary logistic regression identified baseline and CGA-derived factors associated with UI at 6-month post-fracture (*n*=331).

**Results:**

Mean age was 81,4±7,8. Pre-fracture, 45% of men had UI and 12% had FI. One-year mortality was 34%. In multivariable-adjusted Cox regression, FI independently predicted mortality (HR 1.72, 95% CI 1.15–2.58), while UI did not. Other independent predictors included age, polypharmacy, non-independent mobility, poor nutritional status, and delayed urinary catheter removal. At CGA, 47% had UI. Cognitive disorder (OR 3.22, 95% CI 1.63–6.35) and non-independent mobility (OR 3.07, 95% CI 1.58–5.97) were independently associated with UI, as were CGA deficits in cognition, function, and physical ability.

**Conclusion:**

Pre-fracture FI is independently associated with increased 1-year mortality in older men with hip fracture, while UI is closely linked to cognitive and functional decline, emphasizing the syndromic nature of incontinence in these patients. Incontinence assessment including both UI and FI may help identify patients at highest risk of adverse outcomes.

## Key summary points

**Aim:**

To evaluate the prevalence and prognostic implications of fecal and urinary incontinence in older men with hip fracture and identify factors associated with urinary incontinence at post-fracture comprehensive geriatric assessment.

**Findings:**

Incontinence is highly prevalent among older men with hip fracture. Fecal incontinence independently predicts one-year mortality, while urinary incontinence is closely linked to cognitive and functional decline in these patients.

**Message:**

Given its high prevalence and prognostic relevance, both fecal and urinary incontinence should be routinely assessed during comprehensive geriatric assessment of older men with hip fracture.

## Introduction

Hip fracture is one of the most devastating consequences of a fall in an older individual and a harbinger of long-lasting impairments in mobility, independence and quality of life [1]. Although hip fracture is considerably more common in women than in men, outcomes in men are disproportionately poor. Greater bone density and muscle mass confer relative protection against hip fracture in men, yet osteoporosis remains underdiagnosed in this population [2]. When men do sustain a hip fracture, they tend to do so at a younger age than women yet present with higher levels of comorbidity and dependency, are more prone to post-operative complications, and have significantly higher mortality rates during the first year after the fracture [3, 4].

Urinary incontinence (UI), defined as involuntary loss of urine, and fecal incontinence (FI), defined as involuntary loss of feces [5], are prevalent conditions whose frequency increases with age. Among men, UI affects approximately 11% of those aged 60–64 and over one-third of those aged 85 and older [6]. FI is estimated to affect 1 in 12 adults worldwide, although social stigma likely leads to under-reporting [7, 8].The two conditions frequently co-exist, with FI often preceded by UI [9].

In older men, benign prostatic enlargement is a key etiologic risk factor for UI [10]. However, incontinence in older adults is best conceptualized as a geriatric syndrome with multifactorial causes that extend beyond incontinence to other geriatric syndromes, such as falls [11]. Indeed, individuals with UI face up to twice the risk of falls compared to their continent peers [12].

The co-occurrence of incontinence and falls in the same individual exemplifies how compounding geriatric syndromes amplify the risk of disability [13]. In hospitalized older and vulnerable adults, incontinence and falls are the two most encountered geriatric syndromes [14]. Furthermore, care-related factors during hospitalization, such as urinary catheter use and continence aids, have been identified as risk factors for incident incontinence and worse functional recovery [15, 16]. In our previous study of older women with hip fracture, pre-fracture incontinence predicted declining mobility levels during the first-year post-fracture, and co-existing UI and FI predicted transition to more supported living arrangements [17].

Literature on the relationship between incontinence and mortality remains inconsistent. In a large community-based cohort, Jamieson et al. found that FI, but not UI, was independently associated with higher mortality than in continent participants [18]. Among hip fracture patients specifically, Guzon-Illescas et al. [1] demonstrated that incontinence — defined as either UI or FI — independently predicted increased mortality, although the analysis did not distinguish between the two types or between sexes.

Because both incontinence and hip fractures are more common in women, existing research has focused predominantly on women or on mixed-sex samples in which men constitute a minority, leaving a substantial gap in knowledge regarding the prevalence, associated factors, and prognostic significance of incontinence among older men with hip fracture. The aims of this study were therefore, threefold: first, to establish the prevalence of any UI and any FI among older men with hip fracture; second, to identify sociodemographic and clinical factors associated with prevalent UI; and third, to investigate whether UI and FI are associated with increased 1-year mortality in this population.

## Methods

This study was a retrospective analysis of prospectively collected data gathered between September 2007 and January 2019. The study population consisted of 644 older men who sustained their first hip fracture during this period. Patients were aged 65 and over and managed in Seinäjoki Central Hospital, the sole provider of acute surgical care in the Hospital District of Southern Ostrobothnia, with a catchment area of around 200,000 residents. Pathologic and periprosthetic fractures were the only exclusion criteria. All the patients were invited to a comprehensive geriatric assessment (CGA) at our out-patient clinic, which took place in a median time of 6 months (IQR 4–6 months) after the hip fracture.

**Fig. 1:**
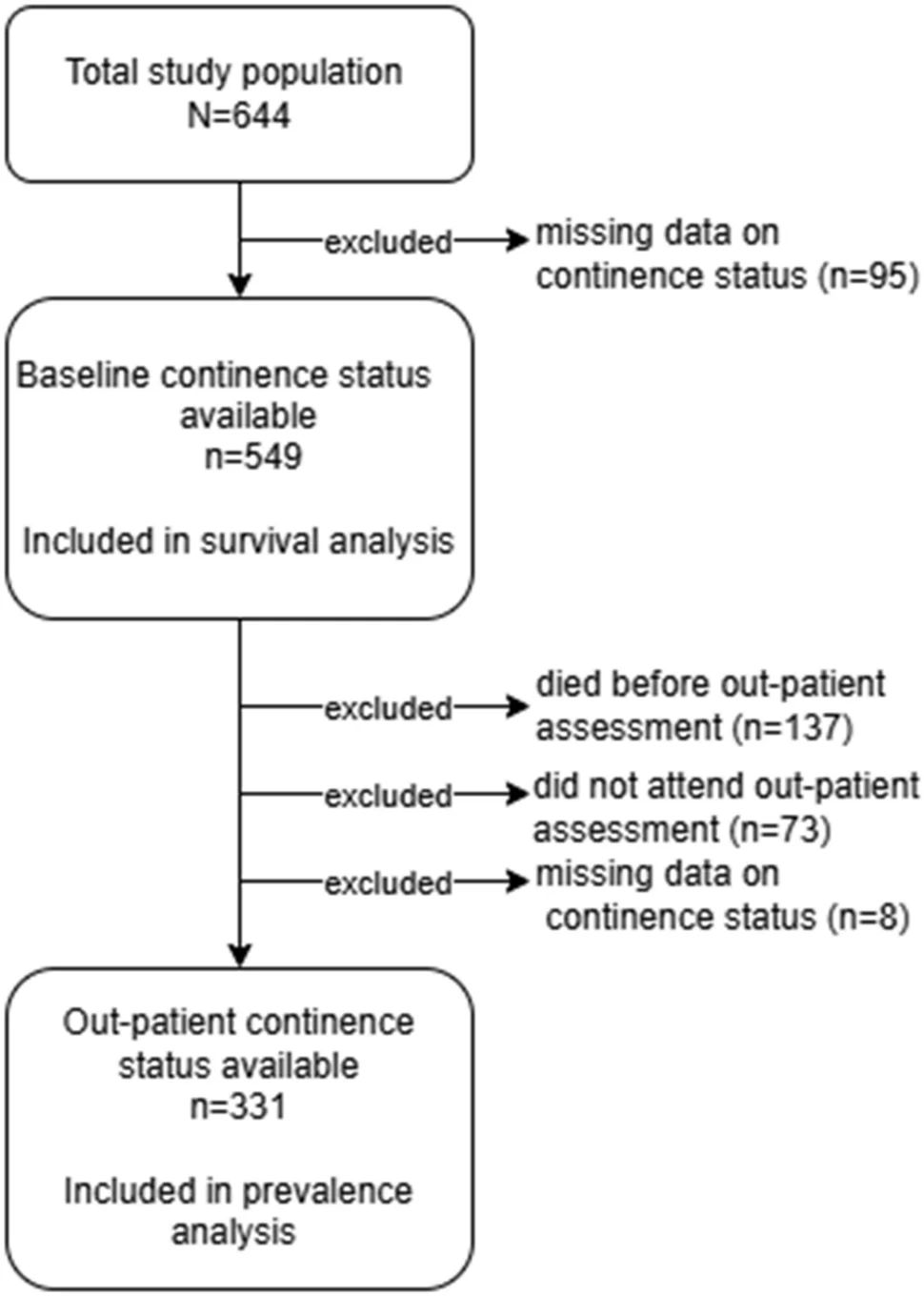
Flow chat of the study population

At baseline, data on UI and FI were missing for 95 patients, leaving 549 men with information on continence status before the fracture. This constituted the sample for the Cox proportional hazard regression analyses examining whether UI or FI predicted 1-year mortality. Of the original cohort, 339 men attended the out-patient CGA at 4–6 months post-fracture, of whom 8 had missing data on continence, yielding a final sample of 331 men for the binary logistic regression analysis of factors associated with prevalent UI. The study population and sample derivation is presented in detail in Fig. 1.

### Study protocol and variables

The study protocol and the variables used in this study have been previously described elsewhere [19–21]. Briefly, a geriatric nurse interviewed the patients or their representatives perioperatively after the hip fracture and retrieved data from the medical records. Data on age, number of prescribed daily medications, known diagnosis of cognitive disorder (yes or no), mobility, and living arrangements were collected. Independent mobility was defined as being able to ambulate independently without assistance. Living arrangements were categorized as either living in own home with or without organized home care or living in assisted living accommodation (no nursing staff present at night) or institution (nursing staff always present).

The preoperative American Society of Anesthesiologists (ASA) risk scores were used to assess general health at the time of hip fracture. There are five classes: (1) healthy person, (2) mild systemic disease, (3) severe systemic disease, (4) severe systemic disease that is a constant threat of life, and (5) a moribund person who is not expected to survive without surgery [22]. ASA score was the indicator of comorbidity in our analysis, and it has been shown to correlate well with Charlson comorbidity index [23]. The removal of urinary catheter was categorized as removed during the hospitalization in the central hospital or later.

At the out-patient clinic, CGA was performed by a multidisciplinary team consisting of a geriatric nurse, a physiotherapist and a geriatrician or a resident in geriatric medicine. Both the patients and their representatives were invited to the out-patient clinic [24]. The geriatric nurse carried out the interview and the assessment of the domains with the selected tools. Cognitive function was measured with the mini mental state examination (MMSE) [25], nutritional status was assessed both at the central hospital as well as at the out-patient clinic using the Mini-Nutritional Assessment Short Form (MNA-SF)[26], depressive mood with the 15-item geriatric depression scale (GDS-15) [27], and instrumental activities of daily living (IADL) by Lawton and Brody [28].

The physiotherapist’s assessment of the patient’s physical performance included the Timed up and go-test (TUG) assessing mobility, balance, and risk of falls [29], the Elderly Mobility Scale (EMS) which represents patient’s mobility and ability to perform basic activities of daily living (ADL) [30], and grip strength as a measure of muscle strength. Grip strength was measured in the stronger hand with a Jamar dynamometer and defined as weakened if less than 27 kg [31]. For statistical purposes, MNA–SF and TUG were split into two categories with points indicating “at risk of being malnourished” and “malnourished” being merged in MNA–SF, and classes 3–5 indicating abnormal result being merged in TUG Table 1, 2, 3.

**Table 1:** Prevalence of urinary and fecal incontinence according to baseline characteristics

		Incontinence					
		UI (*n* = 246)			FI (*n* = 66)		
Baseline characteristic	*N*	*n*	(%)	*p*	*n*	(%)	*p*
*Age*				<0.001			0.089
65–79	212	70	(29)		19	(29)	
80–89	254	125	(51)		32	(49)	
≥ 90	83	51	(21)		15	(23)	
*Diagnosis of cognitive disorder*				<0.001			<0.001
No	392	135	(55)		27	(41)	
Yes	157	111	(45)		39	(59)	
Mobility*				<0.001			<0.001
Independent	313	81	(33)		15	(23)	
Non-independent	235	165	(67)		51	(77)	
Living arrangements*				<0.001			<0.001
Home	412	133	(54)		24	(36)	
Institution	135	113	(46)		42	(64)	

^*^Small number of missing values (*n*=1–2) are not shown in table

Group comparisons between participants with and without urinary incontinence (UI) and with and without fecal incontinence (FI) were performed using Pearson's χ^2^ test or Fisher's exact test, as appropriate

**Table 2:** Distribution of the baseline indicators between alive and deceased individuals (N=549) after 1-year of follow-up

		12-month survival							
		Deceased		Alive					
		*n* = 186		*n* = 363		Age-adjusted		Multivariable-adjusted	
	N	*n*	(%)	*n*	(%)	HR	(95% CI)	HR	(95% CI)
*Age*									
65–79	212	48	(26)	164	(45)	1.00		1.00	
80–89	254	94	(51)	160	(44)	**1.83**	**(1.29–2.60)**	**1.50**	**(1.03–2.19)**
≥ 90	83	44	(24)	39	(11)	**3.11**	**(2.06–4.70)**	**2.32**	**(1.50–3.60)**
*Fracture type*									
Intracapsular	359	127	(68)	232	(64)	1.00		1.00	
Extracapsular	190	59	(32)	131	(36)	0.16	(0.59–1.09)	0.76	(0.55–1.04)
*ASA*									
1–2	78	11	(6)	67	(19)	1.00		1.00	
3–5	454	167	(90)	287	(79)	**2.38**	**(1.28–4.44)**	1.91	(0.96–3.79)
Not known	17	8	(4)	9	(3)	**3.86**	**(1.55–9.61)**	2.44	(0.92–6.45)
*Number of regularly taken medications*									
< 4	86	13	(7)	73	(20)	1.00		1.00	
4–10	345	116	(62)	229	(63)	**2.31**	**(1.30–4.12)**	1.42	(0.76–2.62)
> 10	118	57	(31)	61	(17)	**3.62**	**(1.98–6.63)**	**1.96**	**(1.02–3.77)**
*Diagnosis of cognitive disorder*									
No	392	112	(60)	280	(77)	1.00		1.00	
Yes	157	74	(40)	83	(23)	**1.74**	**(1.29–2.35)**	1.11	(0.80–1.55)
Mobility*									
Independent	313	67	(36)	246	(68)	1.00		1.00	
Non-independent	235	119	(64)	116	(32)	**2.77**	**(2.04–3.76)**	**1.97**	**(1.35–2.89)**
Living arrangements*									
Home	412	115	(62)	297	(82)	1.00		1.00	
Institution	135	71	(38)	64	(18)	**2.00**	**(1.49–2.71)**	1.08	(0.75–1.55)
*MNA-SF before hip fracture*									
Normal (12–14)	263	63	(34)	200	(55)	1.00		1.00	
Poor nutrition (< 12)	203	102	(55)	101	(28)	**2.32**	**(1.69–3.19)**	**1.45**	**(1.02–2.06)**
Not known	83	21	(11)	62	(17)	0.85	(0.64–1.72)	0.67	(0.39–1.14)
*Removal of urine catheter*									
During hospital stay	318	86	(46)	232	(64)	1.00		1.00	
Later	208	85	(46)	123	(34)	**1.54**	**(1.14–2.09)**	**1.50**	**(1.09–2.06)**
Not known	23	15	(8)	8	(2)	**7.17**	**(4.07–12.6)**	**6.35**	**(3.55–11.4)**
*UI before fracture*									
No	303	77	(41)	226	(62)	1.00		1.00	
Yes	246	109	(59)	137	(38)	**1.76**	**(1.31–2.37)**	0.85	(0.59–1.22)
*FI before fracture*									
No	483	147	(79)	336	(93)	1.00		1.00	
Yes	66	39	(21)	27	(7)	**2.38**	**(1.67–3.39)**	**1.72**	**(1.15–2.58)**

Age-and multivariable-adjusted survival with hazard ratios (HR) and 95% confidence intervals (CI) were calculated using Cox regression

Statistically significant results (p<0.05) were bolded

*ASA* American society of anesthesiologists -risk score, *MNA-SF* mini-nutritional assessment short form, *UI* urinary incontinence, *FI* fecal incontinence. *Small numbers of missing values (*n*=1–2) were not shown in table

**Table 3:** Age- and multivariable adjusted associations of baseline variables with prevalent urinary incontinence at the out-patient clinic at six-months among men with hip fracture (*N*=331)

		Continent *n*=174		Urinary incontinent *n*=157		Age-adjusted		Multivariable-adjusted	
	*N*	*n*	(%)	*n*	(%)	OR	(95% CI)	OR	(95% CI)
*Age*									
65–79	151	95	(55)	56	(36)	1.00		1.00	
80–89	254	70	(40)	88	(56)	**2.13**	**(1.35–3.36)**	1.37	(0.80–2.35)
≥ 90	83	9	(5)	13	(8)	2.45	(0.98–6.10)	1.46	(0.53–4.04)
*Fracture type*									
Intracapsular	218	114	(66)	104	(66)	1.00			
Extracapsular	113	60	(35)	53	(34)	0.95	(0.60–1.51)	Excluded^●^	
*ASA*									
1–2	66	49	(28)	17	(11)	1.00		1.00	
3–5	255	122	(70)	133	(85)	**2.55**	**(1.36–4.77)**	1.97	(0.95–4.09)
*Number of regularly taken medications*									
< 4	69	49	(28)	20	(13)	1.00		1.00	
4–10	204	103	(59)	101	(64)	**2.21**	**(1.22–4.03)**	1.20	(0.60–2.39)
> 10	58	22	(13)	36	(23)	**3.76**	**(1.77–8.00)**	1.39	(0.54–3.60)
*Diagnosis of cognitive disorder*									
No	259	158	(91)	101	(64)	1.00		1.00	
Yes	72	16	(9)	56	(36)	**5.07**	**(2.74–9.39)**	**3.22**	**(1.63–6.35)**
Mobility*									
Independent	238	151	(87)	87	(55)	1.00		1.00	
Non-independent	92	23	(13)	69	(44)	**4.76**	**(2.75–8.24)**	**3.07**	**(1.58–5.97)**
Living arrangements*									
Home with/without home care	278	160	(92)	118	(75)	1.00		1.00	
Other than home	51	12	(7)	39	(25)	**4.20**	**(2.09–8.44)**	1.72	(0.76–3.91)
*MNA-SF before hip fracture*									
Normal (12–14)	188	108	(62)	80	(51)	1.00		1.00	
Poor nutrition (< 12)	90	33	(19)	57	(57)	**2.19**	**(1.29–3.70)**	1.28	(0.69–2.40)
Not known	53	33	(19)	20	(13)	0.82	(0.43–1.54)	0.58	(0.28–1.20)
Removal of urinary catheter*									
During hospital stay	218	120	(69)	98	(62)	1.00		Excluded^●^	
Later	105	49	(28)	56	(36)	1.35	(0.84–2.17)		

No urinary incontinence (*n*=174) was the reference

*Small numbers of missing values (*n*≤10) were not shown in table. Statistically significant results (*p*<0.05) were bolded. ^●^ Variables not significant in the age-adjusted analyses were excluded from the final multivariable model

*ASA* American society of anesthesiologists -risk score, *MNA-SF* mini-nutritional assessment short form

UI was defined as any reported involuntary loss of urine (yes or no), and FI as any reported involuntary loss of feces (yes or no), as in our previous works [19, 20], and elicited during the perioperative interview as well as at the out-patient clinic. Data on self-reported constipation (yes or no) and new falls after fracture (yes or no) were also elicited. Considering we expected a substantial proportion of participants to be considerably old and/or cognitively impaired, simple survey-like questions were preferred to using structured questionnaires. The categorization of out-patient domains is presented in Table 4.

**Table 4:** Age- and multivariable-adjusted associations of outpatient CGA domains with prevalent urinary incontinence at 6 months of follow-up with no urinary incontinence as the reference group

		Continent *n*=174		Urinary incontinent *n*=157		Age-adjusted		Multivariable-adjusted	
	*N*	*n*	(%)	*n*	(%)	OR	(95% CI)	OR	(95% CI)
*Age*									
65–79	151	95	(55)	56	(36)	1.00		1.00	
80–89	158	70	(40)	88	(56)	**2.13**	**(1.35–3.36)**	1.31	(0.75–2.29)
≥ 90	22	9	(5)	13	(8)	2.45	(0.98–6.10)	0.91	(0.32–2.63)
MMSE*									
Normal (24–30)	123	89	(51)	34	(22)	1.00		1.00	
Abnormal (< 24)	203	84	(48)	119	(76)	**3.34**	**(2.03–5.48)**	**1.94**	**(1.11–3.39)**
*IADL*									
No difficulties (8)	38	34	(20)	4	(3)	1.00		1.00	
Difficulties (0–7)	281	136	(78)	145	(92)	**8.46**	**(2.90–24.7)**	**3.42**	**(1.10–10.62)**
Not known	12	4	(2)	8	(5)	**15.9**	**(3.21–79.0)**	5.23	(0.76–36.11)
GDS-15*									
Normal (0–6)	262	151	(87)	111	(71)	1.00		1.00	
Depressed (> 6)	59	21	(12)	38	(24)	**2.31**	**(1.27–4.19)**	1.48	(0.74–2.96)
*EMS*									
Normal (14–20)	225	148	(85)	77	(49)	1.00		1.00	
Abnormal (< 14)	87	22	(13)	65	(41)	**5.10**	**(2.88–9.00)**	**2.46**	**(1.25–4.84)**
Not known	19	4	(2)	15	(10)	**6.80**	**(2.17–21.3)**	2.00	(0.45–8.85)
*TUG*									
Normal (1–2)	101	77	(45)	24	(15)	1.00		1.00	
Abnormal (3–5)	179	84	(49)	95	(61)	**3.33**	**(1.92–5.79)**	**2.07**	**(1.07–3.98)**
Not known	50	12	(7)	38	(24)	**9.38**	**(4.20–20.9)**	3.57	(1.24–10.2)
*Grip strength, stronger hand*									
Normal (≥ 27 kg)	90	62	(36)	28	(18)	1.00		1.00	
Abnormal (< 27 kg)	161	70	(40)	91	(58)	**2.35**	**(1.32–4.16)**	1.33	(0.68–2.61)
Not known	80	42	(24)	38	(24)	1.82	(0.96–3.44)	0.64	(0.29–1.41)
MNA-SF*									
Normal (12–14)	153	96	(56)	57	(36)	1.00		1.00	
Poor nutrition (< 12)	169	75	(43)	94	(60)	**2.04**	**(1.29–3.22)**	0.98	(0.56–1.72)
*Constipation*									
No	133	75	(57)	58	(44)	1.00		Excluded^●^	
Yes	129	56	(43)	73	(56)	1.51	(0.92–2.49)		
Not known	69	43	(25)	26	(17)	0.77	(0.42–1.40)		
*Fecal incontinent*									
No	291	164	(94)	127	(81)	1.00		1.00	
Yes	40	10	(6)	30	(19)	**3.85**	**(1.79–8.27)**	1.49	(0.60–3.71)
*New falls after fracture*									
No	231	129	(74)	102	(65)	1.00		Excluded^●^	
Yes	100	45	(26)	55	(35)	1.47	(0.91–2.38)		

Results are shown by odds ratios (OR) with 95% Confidence intervals (CI). Statistically significant ORs (p<0.05) are in bold. *MMSE* mini mental state examination, *IADL* instrumental activities of daily living, *GDS-15* geriatric depression scale, *EMS* elderly mobility scale, *TUG* timed up and go –test, *MNA-SF* mini-nutritional assessment short form. *Small numbers of missing values (*n*≤10) were not shown in table. ^●^Variables not significant in the age-adjusted analyses were excluded from the final multivariable model

The dates of death were provided by the National population register center and extracted from the electronic patient medical records. There were no losses to mortality follow-up.

### Ethical statement

All participants or their representatives (legal guardian or next of kin) gave written informed consent. The original study design was reviewed and approved in a meeting of the Ethics Committee of the Hospital District of Southern Ostrobothnia, Seinäjoki, Finland on the 1st of November 2007.The study complies with the ethical standards of the 1964 Declaration of Helsinki and its later amendments.

### Statistical analyses

Group comparisons between participants with and without urinary incontinence (UI) and with and without fecal incontinence (FI) were performed using Pearson's χ^2^ test or Fisher's exact test. Age- and multivariable-adjusted Cox proportional hazard regression models were used to examine associations of UI, FI, and other baseline predictors with 1-year mortality, reported as hazard ratios (HR) with 95% confidence intervals (CI). The proportional hazards assumption was assessed visually using log-minus–log survival plots for all covariates and formally tested for the primary exposure variables UI and FI by including time-dependent interaction terms (covariate × time) in the Cox model. No violations were detected. All baseline variables, described above, were included in the multivariable-adjusted Cox model. Age- and multivariable-adjusted binary logistic regression models were used to examine associations of baseline predictors and out-patient CGA domains with prevalent UI at 6-month post-fracture, reported as odds ratios (OR) with 95% CI. In the logistic regression models, variables that were not statistically significant in the age-adjusted analyses were excluded from the final multivariable-adjusted models. We also examined the interaction between UI and FI, but too few cases of FI occurred to report reliable results. Participants with missing values were retained in the analyses by treating missing values as a separate category. No imputation of missing data was performed. IBM SPSS Statistics version 31.0 (SPSS Inc., Chicago, Illinois) was used for all analyses. All tests were two-sided and *p*-values <0.05 were considered statistically significant.

## Results

Mean age of the participants was 81,4 years (SD=7,8). Before hip fracture, 246 (45%) of the men had UI, and 66 (12%) FI, respectively. Out of 66 men with FI, 59 had co-existing UI. Compared with continent participants, those with UI or FI were more likely to have a cognitive disorder, impaired pre-fracture mobility, and to live in an institution. Participants with UI were also older than their continent counterparts (Table 1). After 1 year of follow-up, 186 (34%) of the men had died. Mean survival time showed a stepwise decline with worsening continence status: continent men survived a mean of 312 days (95% CI 299–326), compared to 269 days (95% CI 248–290) for men with UI only and 217 days (95% CI 177–257) for men with FI with or without co-existing UI.

Among the 331 surviving men who attended the out-patient CGA, 157 (47%) had UI and 40 (12%) had FI, respectively.

In the age-adjusted Cox proportional hazards model, UI (HR 1.83, 95% CI1.29–2.60) and FI (HR 3.11, 95% CI 2.06–4.70) were associated with 1-year all-cause mortality, as well as all the other baseline predictors except for fracture type. FI remained independently associated with 1-year mortality in the multivariable-adjusted model (HR 1.72, 95% CI 1.15–2.58) while UI lost its predictive power. Other significant factors that predicted 1-year mortality in the multivariable-adjusted model were age between 80 and 89 (HR 1.50, 95% CI 1.03–2.19), age 90 and over (HR 2.32, 95% CI 1.50–3.60), 10 or more daily medications (HR 1.96, 95% CI 1.02–3.77), non-independent mobility (HR 1.97, 95% CI 1.35–2.89), poor nutrition according to MNA-SF (HR 1.45, 95% CI 1.02–2.06) as well as late removal of urinary catheter (HR 1.50, 95% CI 1.09–2.06) (Table 2).

In the age-adjusted binary logistic regression analyses, all the baseline variables except for fracture type and removal of urinary catheter were independently associated with UI at the out-patient clinic. In the final multivariable-adjusted model, known diagnosis of cognitive disorder (OR 3.22, 95% CI 1.63–6.35) and non-independent mobility (OR 3.07, 95% CI 1.58–5.97) were independently associated with UI at the out-patient clinic (Table 3). The corresponding results for the associations of the domains of the CGA were the following: All the variables except for constipation and new falls after fracture were independently associated with UI at the out-patient clinic, and MMSE (OR 1.94, 95% CI 1.11–3.39), IADL (OR 3.42, 95% CI 1.10–10.62), EMS (OR 2.46, 95% CI 1.25–4.84) and TUG (OR 2.07, 95% CI 1.07–3.98) remained independently associated with UI in the multivariable-adjusted analyses (Table 4).

## Discussion

This study establishes FI as an independent predictor of all-cause mortality among older men with hip fracture. Moreover, men with limited mobility and a known diagnosis of cognitive disorder were three times more likely to have UI at the out-patient clinic 6-month post-fracture compared to those that did not have these conditions. UI at the out-patient clinic was also associated with several impairments in the domains of the CGA.

UI and FI were prevalent conditions in our study cohort. The pre-fracture prevalence of UI in this study (45%) is in accordance with previous research on mixed-sex hip fracture samples, in which prevalence has ranged between 44 and 62%, although men have constituted a minority in these studies [32, 33]. Similarly, the prevalence of FI (12%) is consistent with two previous mixed-sex hip fracture studies reporting rates between 9 and 14% [34, 35].

FI independently predicted 1-year mortality in the present study. Our results are in accord with Jamieson’s and colleagues who demonstrated that among older community-dwelling men and women with complex needs, FI emerged as an independent predictor of mortality whereas UI did not [18]. Our study cohort resembles that of Jamieson’s and colleagues in that both studies included individuals who were old and frail. The underlying driver of this connection likely is that of more rapid deterioration of physical function in individuals with FI compared to those without. Older adults with FI have been demonstrated to have more rapid decline in physical performance over a 4-year follow-up compared to their continent peers [36]. Mirroring the same phenomenon, FI has been established to be an independent risk factor to falls in older frail individuals [37]. Moreover, in a recent nationally representative study, phenotypic age acceleration, based on an array of clinical biomarkers, was found to partially mediate the association between FI and mortality [38].

In contrast, UI was not independently associated with 1-year mortality after multivariable adjustment, despite a stepwise decline in mean survival time with worsening continence status. This is consistent with our own previous study of older women with hip fracture, in which neither UI nor co-existing UI and FI independently predicted mortality, although a similar stepwise worsening in survival according to worsening continence status was observed [20]. Our findings also concur with broader literature, in which UI has failed to emerge as an independent predictor of mortality after adjustment for comorbidities and frailty [18, 39–41]. Taken together, the current evidence suggests that UI primarily reflects the underlying disability and frailty of the affected individual rather than conferring independent prognostic risk. The other independent predictors of mortality—high age, polypharmacy, malnutrition, impaired mobility and delayed removal of urinary catheter – align with our prior findings and the broader literature [1, 20, 42, 43]. As a care-related factor, unnecessary urinary catheterization and prolonged catheter use should be minimized, consistent with current guidelines. However, because prolonged catheter use may also reflect greater frailty and clinical complexity, prospective interventional studies are needed to determine whether optimized catheter management improves outcomes [44].

A known cognitive disorder before hip fracture as well as an abnormal MMSE result at the out-patient clinic were independently associated with UI. Cognitive impairment and incontinence are closely intertwined, with rates of incontinence being substantially higher among cognitively impaired individuals compared to those without [45]. Cognitive impairment is further associated with physical frailty, falls [46, 47] and an elevated risk of hip fracture [48]. Among hip fracture patients, known or newly identified cognitive impairment has been reported in up to half of cases [24], a factor that may partly explain the high rates of incontinence in this population [32]. From a clinical standpoint, cognitively impaired hip fracture patients tend to present to the emergency department with greater delay and in a worse clinical state and are at increased risk of complications and mortality [49]. As men with hip fracture already carry a disproportionately high burden of mortality and complications compared to women [3, 4], the coexistence of cognitive impairment and UI may signal a particularly high-risk group.

Impaired mobility at baseline was the other factor independently associated with UI at the outpatient clinic. This finding was further supported by the independent associations observed between UI and abnormal EMS, TUG and IADL scores. Together, these measures capture overlapping but distinct dimensions of physical function: EMS encompasses both mobility and basic ADLs [30], TUG reflects mobility and falls risk [29], and IADL captures the ability to perform instrumental daily tasks [28]. Collectively, these associations underscore the close link between UI and broader physical and functional decline in this population, corroborating previous findings that have identified impaired mobility and self-care as key risk factors for UI among older men in community settings [10, 50]. In the hip fracture context, Sørbye and Grue demonstrated that older hip fracture patients with pre-fracture UI were more likely to experience decline in ADL during the first-year post-fracture [33]. Among older women with hip fracture, our own prior studies have demonstrated similar associations between functional and physical disability and UI [19, 21], as well as a bi-directional relationship between UI and mobility during first-year post-fracture [17, 20]. Our present findings suggest that similar associations might exist among older men with hip fracture. However, the high mortality rate in this cohort meant that the number of survivors available for analysis of incident UI was insufficient to permit meaningful statistical evaluation, precluding a longitudinal examination comparable to our previous work in women.

This study was based on a sizeable, prospectively collected cohort of older men with hip fracture. The study population is representative of real-world clinical practice, with pathological and periprosthetic fractures as the sole exclusion criteria. All eligible patients were invited to participate regardless of their place of residence or cognitive status, with notably nearly one-third having a cognitive disorder and a quarter residing in institutional care at baseline. This inclusive approach enhances the generalizability of our findings. Data were systematically collected by a dedicated geriatric nurse, and well-established, validated tools were used in the out-patient CGA. There were no losses to mortality follow-up.

Although the data were collected prospectively, the study design was retrospective, which accounts for several limitations. Incontinence was assessed using brief structured questions rather than validated continence questionnaires. While this approach was chosen to maximize response rates given the anticipated high levels of cognitive impairment, it did not capture information on the specific types of incontinence or the frequency and severity of symptoms. Furthermore, we did not collect data on the impact of incontinence on participants' quality of life. ASA score served as the only indicator of comorbidity burden, though it has been shown to correlate well with the Charlson comorbidity index among HF patients [23]. Notably, no data were available on pre-existing urological conditions or treatments, such as benign prostatic enlargement, lower urinary tract symptoms, prior prostate surgery, or the use of urological medications. These factors may influence the occurrence of UI and could have contributed to residual confounding. The data collection period spanned over a decade, during which time advances in hip fracture treatment and rehabilitation practices may have influenced outcomes. Finally, the high 1-year mortality rate (34%) introduces survival and selection bias, as the men who died before the out-patient CGA were likely to be in the poorest health with a high prevalence of incontinence, potentially leading to underestimated prevalence rates at the out-patient visit and limiting the sample available for the logistic regression analysis. For these reasons, FI could not be reliably modeled as an outcome and was instead included as an independent variable in the UI model.

## Conclusions

FI is independently associated with increased mortality, and UI is closely associated with cognitive impairment and functional decline in older men with hip fracture. Routine continence assessment including both FI and UI in this population may help identify those at greatest risk of adverse outcomes and guide targeted interventions.

## Data Availability

The datasets generated and analysed during the current study are not publicly available due to limitations of ethical approval involving the patient data and anonymity but are available from the corresponding author on reasonable request.
